# Heart Failure-Inducible Gene Therapy Targeting Protein Phosphatase 1 Prevents Progressive Left Ventricular Remodeling

**DOI:** 10.1371/journal.pone.0035875

**Published:** 2012-04-27

**Authors:** Yosuke Miyazaki, Yasuhiro Ikeda, Kozo Shiraishi, Shizuka N. Fujimoto, Hidekazu Aoyama, Koichi Yoshimura, Makoto Inui, Masahiko Hoshijima, Hideko Kasahara, Hiroki Aoki, Masunori Matsuzaki

**Affiliations:** 1 Department of Medicine and Clinical Science, Division of Cardiology, Yamaguchi University Graduate School of Medicine, Ube, Japan; 2 Department of Surgery and Clinical Science, Yamaguchi University Graduate School of Medicine, Ube, Japan; 3 Department of Pharmacology, Yamaguchi University Graduate School of Medicine, Ube, Japan; 4 Department of Medicine, University of California San Diego, La Jolla, California, United States of America; 5 Department of Physiology and Functional Genomics, University of Florida, Gainesville, Florida, United States of America; 6 Cardiovascular Research Institute, Kurume University, Kurume, Japan; I2MC INSERM UMR U1048, France

## Abstract

**Background:**

The targeting of Ca^2+^ cycling has emerged as a potential therapy for the treatment of severe heart failure. These approaches include gene therapy directed at overexpressing sarcoplasmic reticulum (SR) Ca^2+^ ATPase, or ablation of phospholamban (PLN) and associated protein phosphatase 1 (PP1) protein complexes. We previously reported that PP1β, one of the PP1 catalytic subunits, predominantly suppresses Ca^2+^ uptake in the SR among the three PP1 isoforms, thereby contributing to Ca^2+^ downregulation in failing hearts. In the present study, we investigated whether heart-failure-inducible PP1β-inhibition by adeno-associated viral-9 (AAV9) vector mediated gene therapy is beneficial for preventing disease progression in genetic cardiomyopathic mice.

**Methods:**

We created an adeno-associated virus 9 (AAV9) vector encoding PP1β short-hairpin RNA (shRNA) or negative control (NC) shRNA. A heart failure inducible gene expression system was employed using the B-type natriuretic protein (BNP) promoter conjugated to emerald-green fluorescence protein (EmGFP) and the shRNA sequence. AAV9 vectors (AAV9-BNP-EmGFP-PP1βshRNA and AAV9-BNP-EmGFP-NCshRNA) were injected into the tail vein (2×10^11^ GC/mouse) of muscle LIM protein deficient mice (MLPKO), followed by serial analysis of echocardiography, hemodynamic measurement, biochemical and histological analysis at 3 months.

**Results:**

In the MLPKO mice, BNP promoter activity was shown to be increased by detecting both EmGFP expression and the induced reduction of PP1β by 25% in the myocardium. Inducible PP1βshRNA delivery preferentially ameliorated left ventricular diastolic function and mitigated adverse ventricular remodeling. PLN phosphorylation was significantly augmented in the AAV9-BNP-EmGFP-PP1βshRNA injected hearts compared with the AAV9-BNP-EmGFP-NCshRNA group. Furthermore, BNP production was reduced, and cardiac interstitial fibrosis was abrogated at 3 months.

**Conclusion:**

Heart failure-inducible molecular targeting of PP1β has potential as a novel therapeutic strategy for heart failure.

## Introduction

Heart failure is a leading cause of morbidity and mortality in developed countries and afflicts more than 55 million people in the United States [1]. Patients with chronic heart failure manifest a progressive form of cardiac dysfunction that is characterized by either reduced left systolic and diastolic ventricular function, or both sides, with ventricular remodeling, arrhythmia, and intracardiac conduction disturbances [2]. Although advances in pharmacological and non-pharmacological therapies, including renin-angiotensin-aldosterone system inhibitors, β-adrenergic receptor blockers and cardiac resynchronization therapy devices, have significantly contributed to improvements in morbidity and mortality over the last decade [1], the current treatments still remain suboptimal. Particularly in elderly patients, heart failure not only is associated with systolic dysfunction, but also diastolic dysfunction, thereby often highly intractable. An increase in the number of elder patients with heart failure is predicted to result in higher health costs due to the necessity of repeated admission of the patients [3]. Therefore, a new therapeutic strategy targeting diastolic cardiac function is needed to help address this situation.

The failing myocardium is characterized by a reduced intracellular Ca^2+^ cycling capacity, phosphorylation imbalances, and altered expression patterns of key proteins in the subcellular microdomains of failing cardiomyocytes [4], [5]. These include hyperphosphorylated ryanodine receptor (RyR), reduced expression of sarcoendoplasmic reticulum Ca^2+^ ATPase (SERCA2a), and hypophosphorylated phospholamban (PLN) in the sarcoplasmic reticulum (SR), resulting in defective intracellular Ca^2+^ cycling and progressive systolic and diastolic dysfunction. Correcting such inefficient Ca^2+^ handling by overexpressing the SERCA2a gene [6], [7] or perturbing its endogenous inhibitor, PLN [8], [9], [10], successfully restored cardiac function and ameliorated heart failure progression in a variety of experimental animal models, clearly demonstrating that SERCA/PLN is a promising therapeutic target. Indeed, adeno-associated virus (AAV) vector mediated SERCA2a gene therapy has been formally started in clinical trials with patients with severe heart failure and showed initial promising results without major complications [11], [12].

It is also postulated that overactivation of protein phosphatase 1 (PP1) is directly associated with inefficient Ca^2+^ cycling by inducing a decreased phosphorylation of PLN in the sarcoplasmic reticulum (SR) of failing hearts [13]. There are lines of evidences showing that PP1 inhibition is an alternative molecular approach for the treatment of heart failure by upregulating intracellular Ca^2+^ cycling [14], [15], [16]. Indeed, we and others have been shown that gene transfer of endogenous PP1 inhibitors, such as constitutive active inhibitor-1 (INH-1c) or inhibitor-2 (INH-2) significantly improved cardiac function and extended survival time in animal models of heart failure [14], [15], [16].

However, there are several concerns regarding clinical applications of the gene therapy approach, including immune response against the therapeutic vector [17], organ specific gene-targeting [18] and optimal regulation of therapeutic gene expression. To our knowledge, there is no optimal vector system available which has a regulation component dependent on disease-severity along with heart muscle-specific gene expression [19]. Therefore, we sought to create a heart-failure-specific gene therapy system using the B-type natriuretic peptide (BNP) promoter [20], RNA polymerase II-mediated short hairpin RNA (shRNA) [21] and an AAV serotype 9 (AAV9) vector [22]. As BNP expression level is reported to be a most reliable marker of disease severity [23] in heart failure, and its basal expression level is quite low in normal hearts and practically negligible in other organs, BNP promoter activity may afford control over therapeutic gene expression in both a disease-severity-dependent- and heart-muscle-specific manner. In addition, AAV9 has the strongest avidity for heart muscle among the serotypes [22], [24]. With regard to the therapeutic target for heart failure, we chose PP1β, the PP1 isoform of which we previously reported that it dominantly suppresses SR-mediated Ca^2+^ uptake [25] via PLN dephosphorylation, and its expression has been reported to be upregulated in failing hearts [26]. In the present study, we investigated whether heart-failure-inducible inhibition of PP1β by AAV9-mediated shRNA gene transfer is beneficial for preventing heart failure progression in muscle LIM protein-deficient (MLPKO) mice cardiomyopathy.

## Materials and Methods

### Animals

All animal protocols were approved by the Yamaguchi University School of Medicine Animal Experiment Committee (institutional permission # 24-014). The animals were treated according to the Guide for the Care and Use of Laboratory Animals published by the US National Institutes of Health (NIH Publication No. 85-23, revised 1996). MLPKO mice of the 129sv strain which were obtained from the University of California San Diego were backcrossed with C57/BL6 strain for at least 10 generations. Four-month-old MLPKO mice with a C57/BL6 background were used in the gene transfer experiments.

### Preparation of adenovirus and adeno-associated virus 9 vectors for shRNA

Adenoviruses- (AdV) and AAV9s- encoding short hairpin RNA (shRNA) were designed to obtain knockdown of mouse PP1β mRNA *in vivo* for the short- and long-term, respectively. Briefly, self-annealing oligonucleotides for PP1βshRNA and negative control sequence (NCshRNA) were designed using a web-based tool from Invitrogen (Carlsbad, CA), and incorporated into AdV and AAV9 vectors harboring an expression cassette of emerald green fluorescent protein (EmGFP) conjugated to the insertion site of shRNA in a microRNA(miR) fragment. In AAV9 vectors, a heart failure inducible gene expression system was employed using the B-type natriuretic protein (BNP) promoter conjugated to EmGFP and the miR cassette. The target sense and anti-sense sequences (Table S1) were heated up to 94°C for 4 minutes, gradually cooled down to 37°C for annealing, and inserted into pcDNA6.2-GW/EmGFP-miR (Invitrogen) [27], yielding the expression cassettes of [EmGFP-(PP1β or NC)-shRNA]. The PP1βshRNA- and NCshRNA- expression cassettes flanked by “attB” and “attP” sequences were transferred to the entry-plasmid (Gateway System, Invitrogen) pENTR, by incubation with BP clonase II® and pDONR221, yielding pENTR-6.2-GW/EmGFP-(PP1β or NC)-shRNA plasmids. The shRNA cassettes were further transferred from the pENTR-plasmid to AdV and AAV9 shuttle plasmids by incubation with either LR Clonase II® and the pDC315-CMV-gateway plasmid, or LR Clonase II® and the pZAC2.1 gateway plasmid, yielding pDC315-CMV-EmGFP-PP1βshRNA/pDC315-CMV-EmGFP-NCshRNA, or pZAC2.1-BNP-EmGFP-PP1βshRNA/pZAC2.1-BNP-EmGFP-NCshRNA. The successful incorporation of shRNA cassettes in the AdV and AAV shuttle vectors was confirmed by DNA sequence analysis in Yamaguchi University Gene Research Center. The AdV shuttle plasmids (pDC315-based, Microbix, Toronto, Canada) were co-transfected with the pBHGlox(delta)E1,3Cre plasmid into HEK293 cells, followed by the purification of AdV, as previously described [15], [25]. The BNP promoter sequence [20] was generously provided by Dr. La Pointes of the Henry Ford Hospital, and pZAC2.1 and AAV9 construction plasmids were kindly provided by Dr. James Wilson, the University of Pennsylvania (UPenn). AAV9 vectors encoding the above expression cassette were produced in the vector core facility at Upenn [28]. The AAV9 vector expressing lacZ driven by cytomegalovirus (CMV) promoter was also provided by Dr. James Wilson, UPenn. For the short term analysis, AdV-CMV-EmGFP-PP1βshRNA and AdV-CMV-EmGFP-NCshRNA were used and, for the long term analysis, AAV9-BNP-EmGFP-PP1βshRNA and AAV9-BNP-EmGFP-NCshRNA were utilized.

### Quantification of PP1β expression

Differentiated C2C12 cells were transfected with the AdVs at a MOI of 20, 100 and 500, and cultured for 120 hours (h) at 37°C in 5%CO_2_/95%O_2_ atmosphere. The total RNA of the gene transfected hearts and C2C12 transfected with the AdVs were prepared by using an RNAeasy kit (Qiagen, Hilden, Germany), and the mRNA expression levels were analyzed with real-time RT-PCR (Lightcycler 1.5,Roche). The RT-PCR primer sets for PP1β and GAPDH described in Table S1.

Cells were lysed with an ice-cooled buffer containing (in mM) 25 Tris-HCl (pH 7.4), 1 EDTA, 1 EGTA, 50 NaCl, 1 DTT, 1 Na_3_VO_4_, 1 PMSF, 1% protease inhibitor cocktail (PIC), 1% NP-40 and 0.5% Na-deoxycholate (RIPA buffer). Equal amounts of the protein samples were prepared by adding an LDS buffer (Invitrogen). Samples were heated at 70°C for 5 min, loaded on 10% bis-tris gel (NuPage, Invitrogen), electrophophoresed and transferred to a PVDF membrane. Expression levels were quantified by immunoblotting using PP1β specific antibodies, followed by incubation with a secondary IgG conjugated to horseradish peroxidase (HRP). Chemiluminescence quantification was performed using Supersignal West Femto Substrate (Pierce, Rockford, IL) followed by quantitative image analysis (LAS-4000, Fuji Film, Japan).

### *In vivo* gene transfer of AdV and AAV9

**Figure 1:**
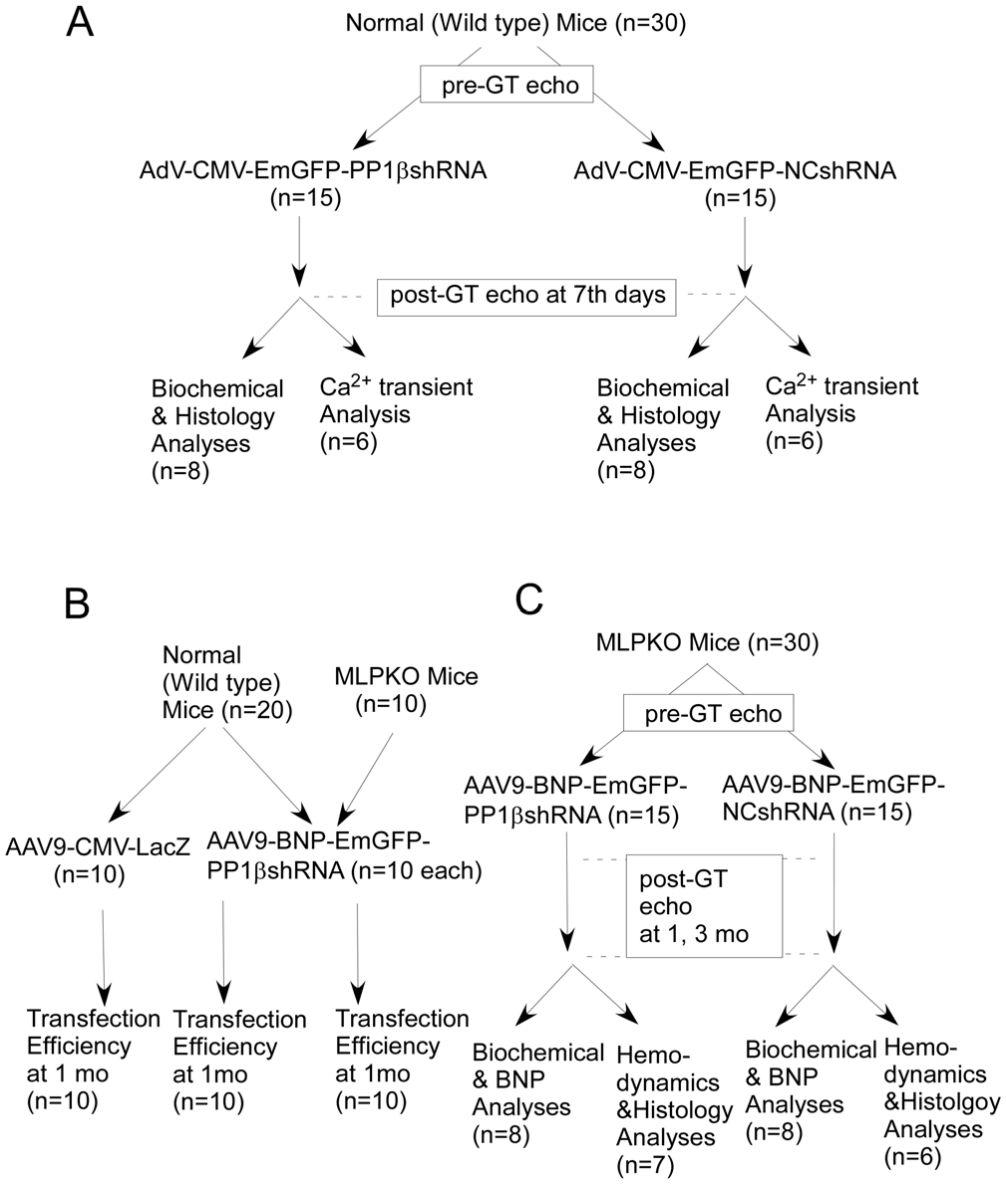
Experimental designs. **A**: The experiment confirming the effect of *in vivo* RNA polymerase II promoter-mediated shRNA in normal mice using AdV vectors. **B**: The experiment confirming AAV9-BNP promoter-mediated gene transfer transfection efficiency at 1 month after gene transfer in normal and MLPKO mice. **C**: The experiment assessing the effects of *in vivo* AAV9-BNP promoter-mediated heart-failure condition-specific shRNA in MLPKO mice cardiomyopathy.

The experimental design is summarized in Figure 1. For the short term analysis of shRNA efficiency, fourteen week-old normal C57/BL6 mice underwent *in vivo* cardiac AdV delivery with direct injection into the left ventricle (LV) (2×10^9^vp per site, 3sites) under general anesthesia with 2% isoflurane, mechanical ventilation and thoracotomy at the 3rd intercostal space, followed by closure of the opened chest and subsequent recovery. On the 7th day after gene transfer, LV systolic function was accessed by echocardiography, followed by biochemical and histological analyses of the heart.

For the long term analysis of cardiac gene transfer, four-month-old wild-type and MLPKO mice underwent AAV9 vector injection via the tail vein (2×10^11^viral particles were injected per mouse). AAV9-CMV-LacZ vector injection was used for analysis of gene transfer efficiency in four-month-old wild type and MLPKO mice (n = 10 in each). One month after AAV9 delivery, AAV9-CMV-LacZ treated heart was excised and transversely sliced at a thickness of 6 µm, followed by β-gal staining, as previously described [15].

### Measurement of cell shortening and the Ca^2+^ transient in isolated mouse cardiomyocytes

One week after intramyocardial injection of AdVshRNA vectors, mouse cardiomyocytes were enzymatically isolated to measure the Ca^2+^ transient and cell shortening. The EmGFP expression positive cardiomyocytes were selected under fluorescent microscopy (488 nm excitation, 510 nm emission filter) and then used to analyze the Ca^2+^ transient and cell shortening, as previously described [9]. The % shortening of the sarcomere and the intracellular Ca^2+^ transient were simultaneously recorded while cells were field-stimulated with 0.5 Hz. Intracellular Ca^2+^ changes were expressed as changes in the fluorescence ratio measured at 340 and 380 nm. The obtained data were analyzed offline by IONOPTIX software (Milton, MA).

### Analysis of SR protein phosphorylation

For analysis of the SR protein phosphorylation level, hearts were homogenized with a buffer containing (in mM) 25 Tris-HCl (pH 7.4), 50 NaCl, 300 sucrose, 1 EDTA, 1 EGTA, 50 NaF, 1 Na_3_VO_4_, 1% Nonidet P-40, 0.5% deoxycholic acid, 0.1% SDS, 0.02% 2-mercaptoethanol and 1% protease inhibitor cocktail (PIC) (Sigma, St. Louis, MO). Protein concentrations were calculated by the Bradford assay, followed by immunoblotting and image analysis with LAS-4000. The phosphorylation levels of phospholamban (PLN) at Ser16 and Thr17, the ryanodine receptor (RyR) at Ser2808 and the cardiac troponin I (TnI) at Ser22 and 23 were normalized to the total protein levels.

### BNP promoter activity assay

The AAV9 shuttle vector plasmid (pZAC2.1-BNP-EmGFP-PP1βshRNA) was transfected into HL-1 cells (generously provided by Dr. W.C. Claycomb, Louisiana State University) using lipofectamine LTX (Invitrogen). Forty eight hours after transfection, cells were stimulated with increasing doses of phenylephrine (2, 5, and 10×10^−4^ M) for 3 days, followed by cell lysis and quantification of BNP mRNA with real time RT-PCR using a primer set of mouse BNP (Table S1) or by the immunoblotting protein expression levels of EmGFP, which represents the BNP promoter activity of the AAV shuttle vector plasmid. The AAV9-vector-transfected hearts were frozen in plastic containers using isopentane precooled with liquid N_2_ and stored at −80°C for further sectioning. Hearts were sectioned at a thickness of 6 µm, fixed with 2% paraformaldehyde in phosphate buffered saline, and immunostained with anti-GFP antibody (ab290, Abcam, Cambridge, UK), followed by chemical reaction with a VECTASTAIN ABC kit (Vector Laboratories, Burlingame, CA) and 3,3′-diaminobenzidine (DAB).

### Assessment of cardiac function

Serial echocardiographic measurements were made at 1 and 3 months after gene transfer, followed by hemodynamic measurement at 3 months before termination. Briefly, the left ventricular diameter and systolic function were serially assessed by a ultrasound machine equipped with a 15 MHz linear probe (HDI-5000 SonoCT, Philips, Netherlands) at 1 and 3 months after gene transfer under general anesthesia using 1.5% isoflurane and spontaneous ventilation.

For hemodynamic analysis, mice were anesthetized with 2% isoflurane, mechanically ventilated, and the right carotid artery was cannulated with a 1F Millar micro-tip catheter placed in the left ventricle (Millar instruments, Houston, TX). Maximum and minimum LV dP/dt and the time constant of relaxation, tau (using the exponential function), were calculated from the LV pressure as described [15].

### Quantification of cardiac interstitial fibrosis

Heart from the basal and mid-ventricular wall were transversely sectioned at a thickness of 6 mm and stained using Heidenhain's trichrome staining method (Azocarmine G, aniline blue, and Orange G). The percentage of the area of interstitial fibrosis in the transverse heart section was automatically calculated in computer-scanned microscopic images using SigmaScanPro 5.0 software (Systat Software Inc., San Jose, CA).

### Antibodies

The following antibodies were obtained from commercially available sources: the antibodies for PP1β (ab16369, ab53315), and PLN (ab2865, clone 2D12: Abcam, Cambridge, UK); phosphorylated-PLN at Ser16, PLN, glyceraldehyde-3-phosphate dehydrogenase (GAPDH) (Chemicon International), phosphorylated-PLN at Thr17, and phoshphorylated-RyR2 at Ser2808 (Badrilla, Leeds, UK); RyR2 (clone C3–33: Sigma-Aldrich, St. Louis, MO), SERCA2a (clone N-19: Santa Cruz Biotechnology, Santa Cruz, CA), cardiac troponin I (cTn-I: clone 19C7), phospho-TnI at Ser22,23 (clone 5E6: Genetex, San Antonio, TX), and GFP(ab290: Abcam).

### Statistical analysis

Comparisons between the two groups were made with Student's t-test. Comparisons between repeated measurements were performed with ANOVA, followed by the post hoc test (the Student-Newman-Keuls method was used to compare the two groups when appropriate). A value of p<0.05 was considered statistically significant. Data are expressed as the mean ± SEM.

## Results

The design of gene transfer using AdV and AAV9 is summarized in Figure 1. Death within 5 days after gene transfer procedure was regarded as improper recovery and these data not incorporated for further analysis (AdV-CMV-EmGFP-PP1βshRNA -treated group; *n* = 1, AdV-CMV-EmGFP-NCshRNA -treated group, *n* = 1). A mouse in the AAV9-BNP-EmGFP-NCshRNA treated group died during the observational period (2 months after gene transfer) possibly due to worsening of heart failure and was not incorporated into further analysis.

### AdV-CMV-EmGFP-PP1βshRNA effectively inhibited PP1β expression both *in vitro* and *in vivo*

**Figure 2:**
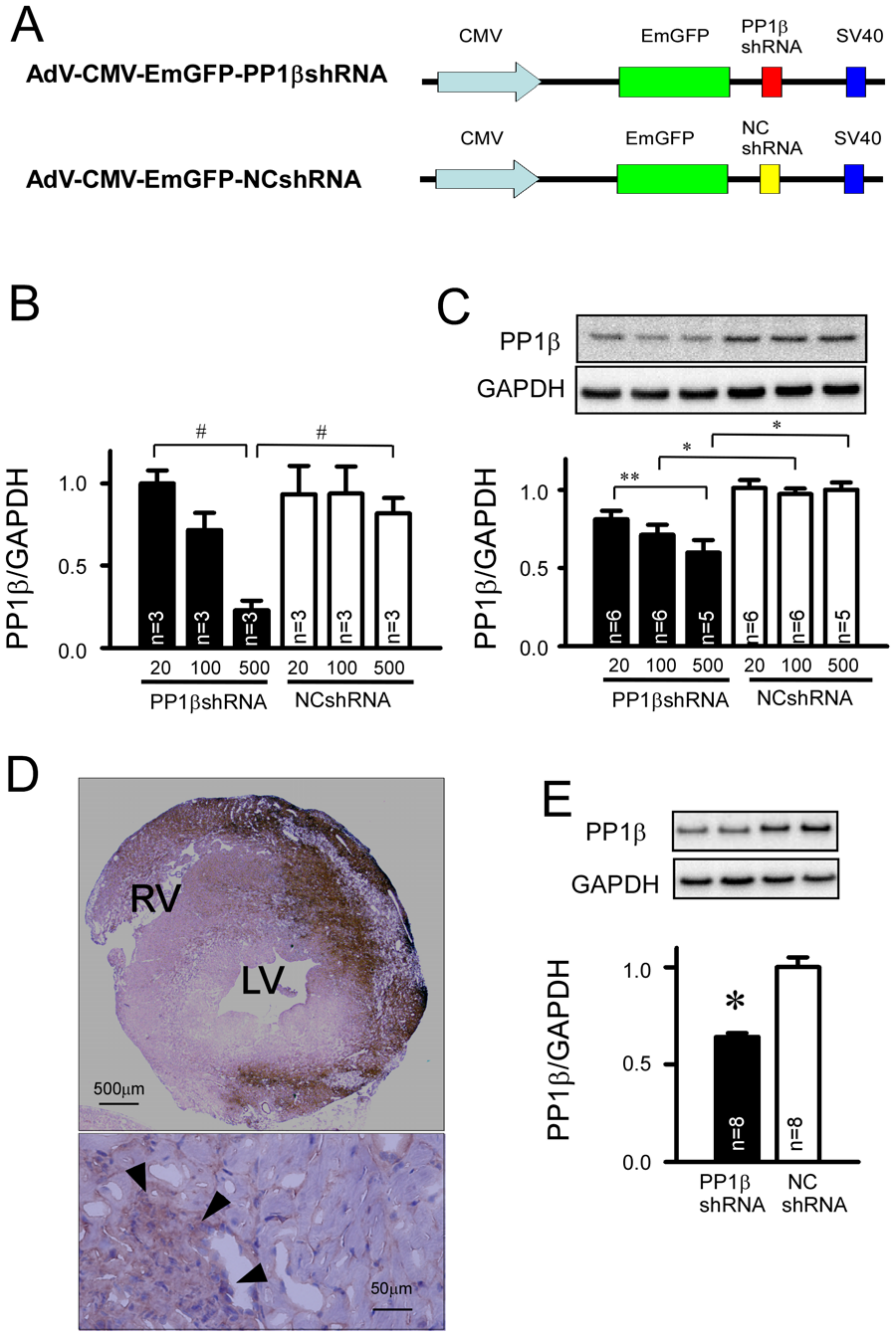
*In vivo* and *in vitro* adenoviral mediated PP1βshRNA significantly reduced expression of PP1β in C2C12 cells and mice hearts. **A**: Vector structure of the adenoviral-PP1βshRNA under the control of the CMV promoter and marker gene expression of EmGFP. The negative control sequence of miR was used as a shRNA control sequence (NCshRNA). **B** and **C**: Efficiency of AdV-CMV-EmGFP-PP1βshRNA assessed by real-time RT-PCR (left panel) and immunoblotting of PP1β (right panel) at increasing MOIs of transfections 5 days after adenoviral infection in differentiated C2C12 cells. **D**: Immunostaining of GFP in the transverse heart section at 7 days after direct adenoviral injection, showing the representative transfection efficiency. The lower panel shows the site of local inflammation (indicated by arrows) 7 days after adenoviral gene transfer. **E**: Immunoblotting of PP1β from LV homogenates and a summary of quantitative image analysis normalized by the amount of GAPDH expression. “*” indicates p<0.05 vs. control. (n = 8 in the PP1βshRNA group, n = 8 in the NCshRNA group).

First, the AdV-PP1βshRNA induced an approximate 70% reduction in the mRNA level (Fig. 2A and B) and 40% reduction in the protein expression level (Fig. 2C) at a MOI of 500 in C2C12 cells compared with that of AdV-NCshRNA. Seven days after gene transfer into the *in vivo* mouse hearts, approximately 40% of the transverse myocardium exhibited GFP-positive immunostaining (Fig. 2D). There were local inflammatory reactions in the GFP-positive myocardial area, probably due to immune reaction against adenoviral vector [29]. In the AdV-CMV-EmGFP-PP1βshRNA injected group, the expression level of the PP1β protein was reduced by 30% compared to that of control vectors (Fig. 2E).

### AdV-PP1βshRNA resulted in enhanced SR Ca^2+^ cycling and increased PLN phosphorylation

**Figure 3:**
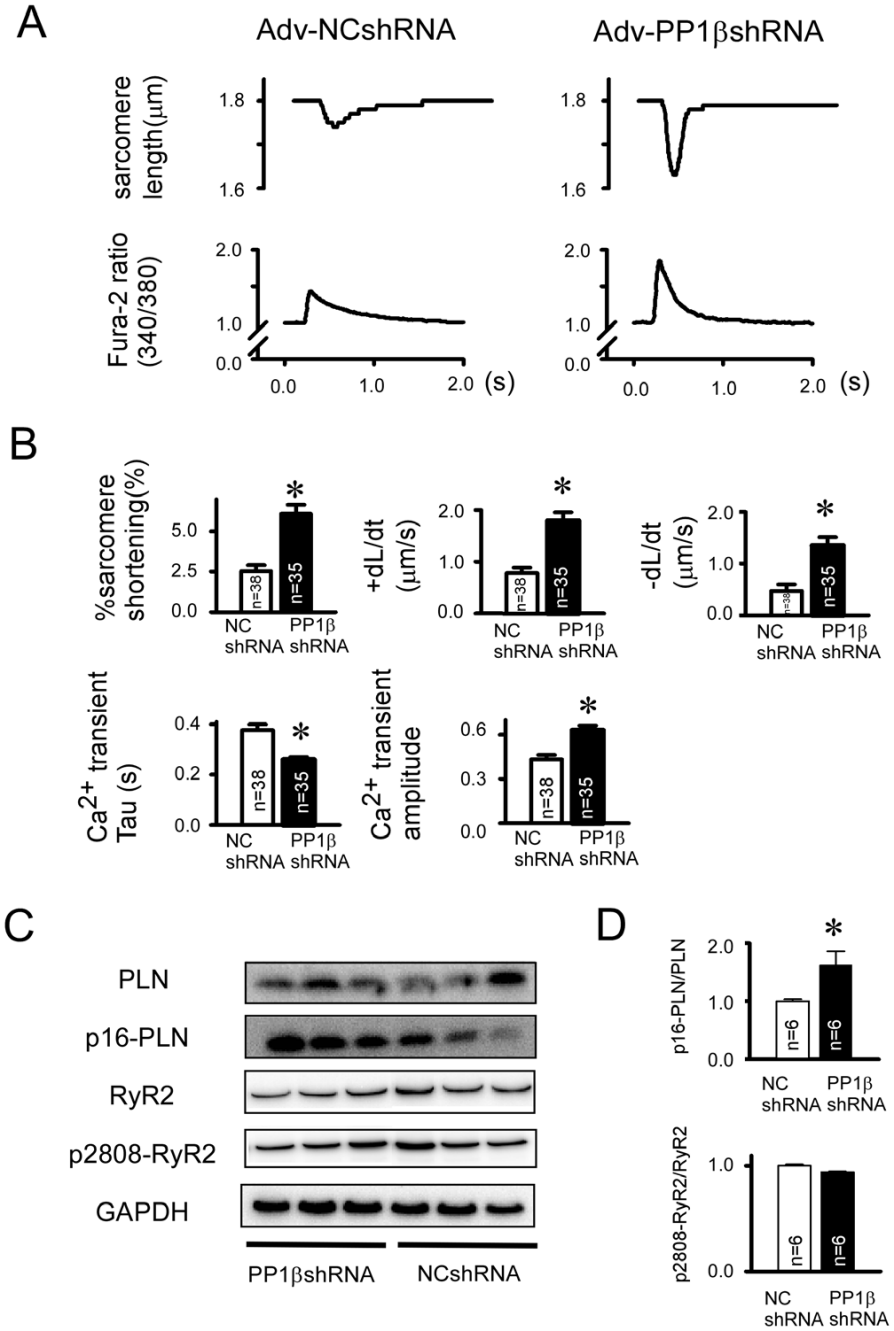
*In vivo* adenoviral PP1βshRNA significantly augmented the Ca^2+^ transient and % sarcomere shortening in AdV-PP1βshRNA mice cardiomyocytes. Cardiomyocytes were isolated from the mouse hearts 7 days after gene transfer, followed by an analysis of sarcomere shortening and the Ca^2+^ transient using the Ionoptix system with fura-2 AM dye. Sarcomere length was calculated in a real-time manner using the SarcLen software of the Ionoptix system. **A**: Representative tracing of sarcomere shortening and Ca^2+^ transient in enzymatically isolated cardiomyocytes from the mouse heart 7 days after adenoviral transfection. **B**: Summaries of the analysis in sarcomere shortening and Ca^2+^ transients; namely, % sarcomere shortening (%SS), the maximum/minimum value of the first derivatives of sarcomere length (+dL/dt, −dL/dt, respectively), the time constant of the Ca^2+^ transient decay slope (Ca^2+^ transient Tau), and the amplitude of the Ca^2+^ transient (Ca^2+^ transient amplitude). “*” indicates p<0.01 vs. NCshRNA control group. (n = 38 in the NCshRNA group, n = 35 in the PP1βshRNA group). **C**: Immunoblots of the key SR phosphoproteins and the phospholylation levels 7 days after gene transfer. Note that the phosphorylation levels of PLN at Ser16 was augmented in the PP1βshRNA treated group compared with that of the NCshRNA treated group. **D**: Summaries of the phospholylation levels of PLN at Ser16 and RyR at 2808 in each group. “*” indicates p<0.05 vs. the NCshRNA treated group. (n = 6 in the NCshRNA group, n = 6 in the PP1βshRNA group).

As shown in the representative tracing of Figure 3A, the AdV-PP1βshRNA treated cardiomyocytes exhibited marked enhancement in the transient Ca^2+^ level and % shortening of the sarcomere length (%SS). There were significant increases in the %SS, maximum/minimum first derivative of sarcomere length (+dL/dt, −dL/dt, respectively), time constant of Ca^2+^ transient decay slope (Tau) and amplitude of the Ca^2+^ transient compared to those of the control vector (Fig. 3B). In heart homogenates, the phosphorylation level of PLN at Ser16 in the PP1βshRNA treated hearts was significantly enhanced compared with those receiving NCshRNA treatment (Fig. 3C). On the other hand, there were no significant changes in the phosphorylation levels of the RyR at Ser2808. Moreover, there were no significant changes in the phosphorylation levels of either PLN at Ser17 or cardiac troponin I at Ser22/Ser23. Furthermore, the protein expression levels of SERCA2a, PP1α and PP1γ was not significantly altered in these two groups (Fig. S1).

### A partial reduction of PP1β is sufficient to enhance LV contractility in the normal mouse heart

One week after adenoviral injection, LV systolic function in the AdV-PP1βshRNA-treated heart showed a significant increase in LV fractional shortening (%FS) without changes in LV end-diastolic diameter or heart rate. These data indicate that a partial reduction of PP1β by PP1βshRNA is sufficient to enhance cardiac contractility by augmenting SR Ca^2+^ cycling in normal mice (Table S2).

### AAV9- mediated inducible PP1βshRNA was only activated in the cardiomyopathic hearts and partially reduced the PP1β expression level

**Figure 4:**
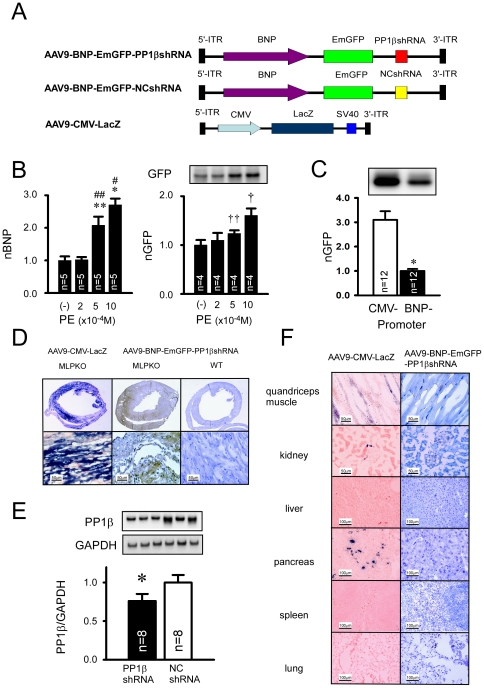
AAV9- mediated heart-failure-inducible PP1βshRNA and partial reduction in PP1βexpression. **A**: Vector structure of the adeno-associated virus 9 (AAV9) -PP1βshRNA flanked by 3′ and 5′ inverted terminal repeats (ITR) under the control of the brain natriuretic peptide (BNP) promoter and marker gene expressison of EmGFP. The negative control sequence of miR was used as a shRNA control sequence (NCshRNA). AAV9 vector expressing lacZ driven by the CMV promoter was used to assess the *in vivo* transfection efficiency through the tail vein injection of AAV9 gene transfer into the heart. **B**: The expression levels of BNP mRNA and EmGFP protein were dose-dependently increased with phenyrephrine (PE) in the pZAC2.1-BNP-EmGFP-PP1βshRNA (AAV9 vector plasmid) transfected HL-1 cells. “*” indicates p<0.001 vs PE(−),“#” indicates p<0.001 vs PE 1×10^−4^ M, “**” indicates p<0.05 vs PE 2×10^−4^ M, “##” indicates p<0.01 vs PE 2×10^−4^ M, “†” p<0.05 vs PE(−),“††” p<0.05 vs PE 2×10^−4^ M. (nBNP; normalized BNP expression, nGFP; normalized EmGFP expression). **C**: Comparison of EmGFP expression levels with BNP- versus CMV- promoter. The normalized EmGFP expression levels were estimated 48 hours after plasmid transfection of pDC316-CMV-EmGFP-PP1βshRNA and pZAC2.1-BNP- EmGFP-PP1βshRNA in HL-cells. “*” indicates p<0.01 vs the CMV promoter treated group. **D**: Bluo-gal staining of transverse heart sections at the papillary muscle level in AAV9-CMV-LacZ transfected MLPKO mice (left panel), and immunostaining of EmGFP proteins in the AAV9-BNP-PP1βshRNA transfected hearts in MLP knockout mice(middle panel) and wild type mice (right panel). The expression level of EmGFP was clearly detected by an anti-GFP antibody (Abcam) as a brown color in the GFP-immunostaining indicates positive cardiomyocytes. The scale bar indicates 50 µm. **E**: Immunoblotting of PP1β in LV homogenates from the transfected MLP knockout hearts with AAV9-BNP-EmGFP-PP1βshRNA and AAV9-BNP-EmGFP-NCshRNA. The graph under the immunoblot indicates quantitative analysis of PP1β expression normalized by GAPDH. PP1β expression levels were decreased by 25% in the PP1βshRNA treated group. “*” indicates p<0.05 vs. NCshRNA treated group. (n = 8 in each group). **F**: Bluo-gal staining of AAV-CMV-LacZ injected mice organs (left column) and immunostaining of GFP inAAV9-EmGFP-PP1βshRNA. There were no detectable levels of the GFP shRNA vector in organs other than the heart, whereas the AAV9-CMV-lacZ vector exhibited a trace expression of lacZ in skeletal muscle, kidney and pancreas. There was no detectable expression in liver, spleen and lung. The scale bar indicates either 50 µm or 100 µm, as indicated in each panel.

Because adenoviral gene transfer showed local inflammatory response (Fig. 2D) and the duration of transgene expression is limited for short term, we switched the gene transfer vector system from AdV to AAV9 for long-term analysis [29]. Namely, we tested whether the AAV9 vector exhibits heart-failure inducible PP1βshRNA in genetic mouse cardiomyopathy (MLPKO mice) (Fig. 1B and C, Fig. 4A).

Induction of the BNP promoter-mediated EmGFP expression and PP1βshRNA were initially tested *in vitro* in a mouse cardiac cell line (HL-1) with increasing doses of an α-adrenergic stimulant, phenylephrine (PE). As shown in the left panel of Figure 4B, PE significantly increased mouse BNP mRNA in a dose-dependent manner. Similarly, the EmGFP protein expression driven by the human BNP promoter in the AAV9 vector was also significantly augmented in a PE-dose-dependent manner (Fig. 4B right panel). The EmGFP expression level with the BNP promoter was approximately one-third compared with that of the CMV promoter in *in vitro* HL-1 cells with 500 µM phenylephrine (Fig. 4C).

Single tail vein injection of the AAV9 vector (2×10^11^ vp) expressing lacZ with the CMV promoter yielded more than 80% positive β-gal-staining myocardium in transverse heart sections (n = 10) from the MLPKO mice at 1 month, confirming the relatively higher infectivity of AAV9 in the heart compared to the other organs (Fig. 4D left panel, Fig. 4F left panels), as previously described [30]. Next, the same amount of the AAV9 (2×10^11^ vp) expressing EmGFP-NCshRNA was injected through the tail vein. One month after gene transfer, a robust expression of EmGFP throughout the transverse heart section was detected in the MLPKO cardiomyopathic heart (Fig. 4D middle), whereas no significant EmGFP expression was detected in the wild type heart (Fig. 4D, right panels).

Furthermore, AAV9-BNP-EmGFP-NCshRNA treated MLPKO mice did not show any EmGFP expression in other organs, including the skeletal muscle, kidney, liver, pancreas, spleen, and lung (Fig. 4F), whereas the AAV-CMV-lacZ treated mice showed trace expression of β-gal positive staining in the skeletal muscle, kidney, and pancreas.

Consequently, PP1βprotein expression was reduced by approximately 25% at 3 months after gene transfer in AAV9-BNP-EmGFP-PP1βshRNA infected MLPKO heart homogenates compared with that of AAV9-EmGFP-NCshRNA treated hearts (n = 8 in each group). Interestingly, the relative amount of PP1β reduction was somewhat similar to the amount of the increase in PP1β expression in the untreated MLPKO hearts compared with that of wild-type mice (Fig. S2).

These data indicate that BNP promoter-driven EmGFP expression is predominantly regulated only in the failing heart, not the normal heart, thereby contributing to the suppression of PP1β protein expression in the failing hearts. Indeed, in MLPKO mice of the same age, there was a dramatic increase in mouse BNP mRNA in the MLPKO mouse heart compared with the wild type (Fig. S3), supporting the notion that PP1βshRNA was only activated in the failing MLPKO hearts.

### AAV9-BNP-promoter-mediated PP1β shRNA significantly improved LV systolic and diastolic function in MLP KO mice

**Figure 5:**
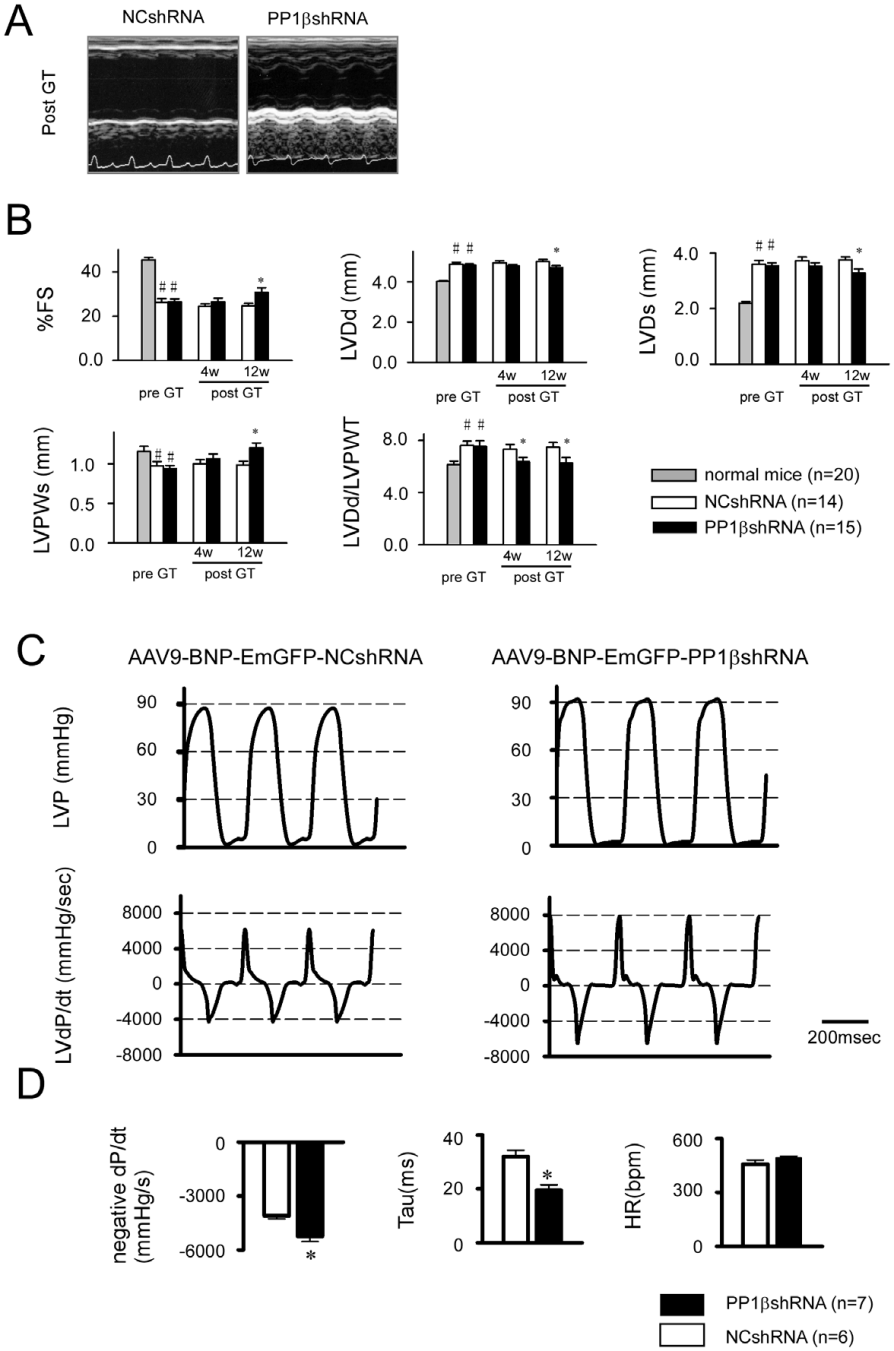
AAV9-mediated heart-failure-inducible PP1βshRNA improved systolic and diastolic function and inhibited LV remodeling. **A**: Representative M-mode echocardiographic tracings 12 weeks after AAV9-BNP-PP1βshRNA treated MLP knockout mice compared with that of AAV9-BNP-NCshRNA treated mice. **B**: Serial cardiac function evaluated by echocardiogram after gene transfer. “*” indicates p<0.05 vs the NC group post GT after 12 weeks. “#” indicates p<0.05 vs. an age-matched wild type mouse. **C**: Representative tracing of LV pressure and dP/dt in the PP1βshRNA and NCshRNA treated groups. The scale bar indicates 200 msec. **D**: Summaries of the hemodynamic data analysis. “*” indicates p<0.05 vs. the NCshRNA treated group, (n = 7 in the PP1βshRNA treated group, n = 6 in NCshRNA treated group.).

At the time of AAV9 injection, MLPKO mice exhibited a marked reduction in the % fractional shortening (%FS) of the LV, as well as dilated LV end-diastolic and end-systolic diameter (LVDd and LVDs, respectively), thinner LV posterior wall thickness (LVPWs), and increased wall stress index (LVDd/LVPWT), compared with that of their wild-type littermates (Fig. 5A and B). The AAV9-BNP-EmGFP-PP1βshRNA treated group exhibited significantly improved %FS, reduced LVDd, and inhibited thinning of LVPWs at 3 months after gene transfer compared with the AAV9-BNP-NCshRNA treated group (Fig. 5A and B). Furthermore, the wall stress index was significantly ameliorated throughout the experimental period in the PP1βshRNA treated group compared with the NCshRNA treated group (Fig. 5B).

As shown in Figure 5C, the PP1βshRNA-treated mice exhibited a dramatically improved LV pressure tracing pattern; namely, the elevated LV end-diastolic pressure was mitigated, and the first derivative curve of LV pressure during diastole exhibited a sharp and convex shape, whereas that of the NCshRNA treated mice exhibited a dull and concave shape (Fig. 5C). As summarized in Figure 5D, AAV9-BNP-PP1βshRNA significantly improved minimum (negative) LV dP/dt and Tau (the time constant of isovolumic LV pressure fitted by exponential function) compared with the AAV9-NCshRNA treated hearts. There was also a tendency of improved maximum (positive) dP/dt in the AAV9-BNP-PP1βshRNA treated MLPKO hearts, but it did not attain statistical significance. These data indicate that AAV9-BNP mediated PP1βshRNA mainly ameliorated LV diastolic function and helped prevent adverse left ventricular remodeling in MLP KO mice throughout the experimental period.

### AAV9-BNP-PP1βshRNA mediated changes in biochemical parameters

**Figure 6:**
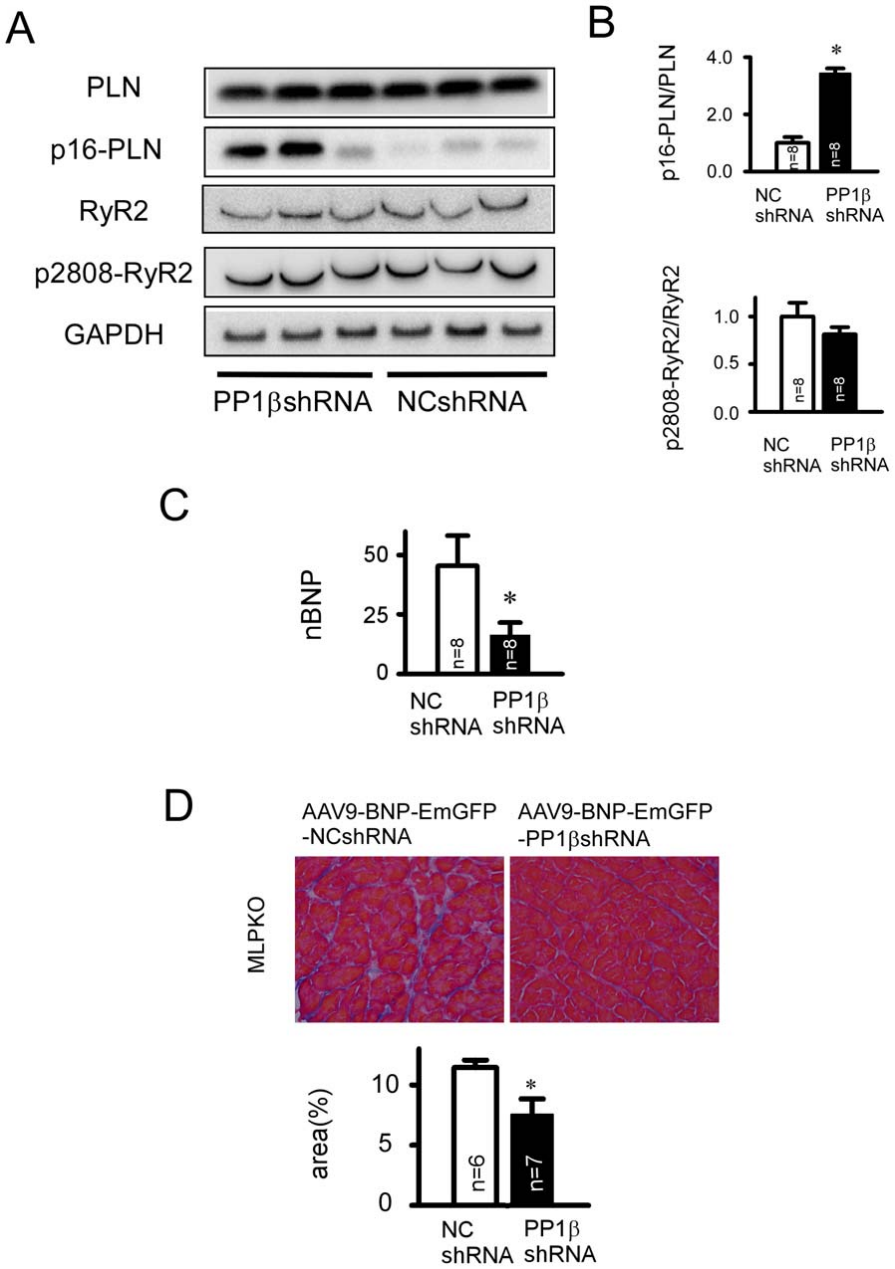
AAV9-mediated heart-failure-inducible PP1βshRNA increased PLN phosphorylation at Ser16, reduced BNP expression, and ameliorated cardiac interstitial fibrosis. **A**: Immunoblots of the key SR phosphoproteins and analysis of the phosphorylation levels by using phosphospecific antibodies in LV homogenates at 3 months after gene transfer. **B**: Summaries of the phospholylation levels of PLN at Ser16 and RyR at Ser2808. “*” indicates p<0.05 vs. the NCshRNA treated group. (n = 8 in PP1βshRNA treated group and n = 8 in NCshRNA treated group). **C**: Expression analysis of normalized BNP (nBNP) using real-time RT-PCR from the AAV9 shRNA transfected heart tissue. “*” indicates p<0.05 vs. the NCshRNA treated group. (n = 8 in the NCshRNA treated group, n = 8 in the PP1βshRNA treated group). **D**: Representative images of Heidenhain's trichrome staining in the AAV9-BNP-EmGFP-NCshRNA- andAAV9-BNP-EmGFP-PP1βshRNA treated hearts at 3 months after gene transfer. The lower graph shows the quantitative image analysis of percentage of the area of interstitial fibrosis. “*” indicates p<0.05 vs. the NCshRNA treated group. (n = 6 in NCshRNA treated group and n = 7 in PP1βshRNA treated group).

In the heart homogenates, the phosphorylation levels of PLN at Ser16 in the AAV9-BNP-EmGFP-PP1βshRNA treated hearts was significantly increased compared with the NCshRNA treated one (Fig. 6A). As similarly observed in the AdV transfection experiment, there were no changes in the phosphorylation levels of the RyR at Ser2808, and no changes in the expression levels of PLN or RyR (Fig. 6 A and B).

Moreover, the AAV9-BNP-PP1βshRNA-treated hearts significantly reduced mouse BNP mRNA expression compared with the AAV9-BNP-NCshRNA treated hearts (Fig. 6C). Cardiac interstitial fibrosis was significantly reduced by BNP-PP1βshRNA compared with NCshRNA. There was no significant changes in the expression levels of the other PP1 isoforms, SERCA2a, or cardiac troponin I (Fig. S4).

## Discussion

In the present study, we demonstrated that AdV vector-mediated PP1βshRNA treatment dramatically augmented SR Ca^2+^ cycling in normal mice cardiomyocytes. Furthermore, AAV9 vector-mediated heart failure-inducible PP1βshRNA treatment prominently improved LV diastolic function, and mitigated progressive left ventricular remodeling in MLPKO mice, an animal model which mimics human dilated cardiomyopathy. In addition, there was a significant reduction in BNP production and cardiac interstitial fibrosis, both of which seem to be coincident with ameliorated cardiac function. To our knowledge, this is the first experimental study that reports a heart-tissue-specific and heart-failure-condition-specific gene therapy for the treatment of heart failure.

The effect of PP1βshRNA on the improved *in vivo* LV function is attributed to increased PLN phosphorylation at Ser16 in the cardiomyopathic hearts. In the previous *in vitro* experimental study, we demonstrated that PP1β was the most significantly involved isoform among the three PP1 catalytic subunits in regulating SR Ca^2+^ uptake. Furthermore, PP1β was found to preferentially interact with the SERCA2a and PLN protein complexes in the longitudinal SR, thereby suppressing SR-mediated Ca^2+^ cycling in cardiomyocytes. As PP1β protein expression has been shown to be upregulated in human heart failure and animal models of cardiomyopathy, shRNA-mediated suppression of PP1β appears to be a promising approach to restore impaired SR Ca^2+^ cycling in the failing heart. Indeed, PP1β expression in failing MLPKO hearts was upregulated by approximately 30% (Fig. S2). BNP promoter driven PP1βshRNA suppressed PP1β expression by approximately 25%, which was similar to the levels of increase in PP1β in the failing myocardium. Thus, this unique gene transfer approach convincingly demonstrates that heart-failure-condition-specific suppression of PP1β is sufficient to improve cardiac function for 3 months in MLPKO cardiomyopathy. In addition, as PP1β is a ubiquitously distributed serine/threonine phosphatase, both the heart-specific and heart-failure specific gene regulation was essential to prevent systemic side effects.

We and others have previously reported that increased PP1 activity was a exacerbating factor in animal models of heart failure [15] as well as human heart failure [31] and that PP1 inhibition by the overexpression of endogenous inhibitors was beneficial to preventing heart failure progression using either viral vector mediated gene transfer study or a transgenic mouse approach [14], [16]. For example, Pathak et al. [32] reported that a constitutive form of INH-1c-overexpressing mouse hearts displayed a hypercontractile phenotype in chronic-pressure-overload- and chronic-isoproterenol-infusion- heart failure models, resulting in rescued heart failure progression. We also reported that AAV2-mediated gene delivery of INH-2 prevented the progression of hamster cardiomyopathy [15]. In contrast, El-Armouche and colleagues [33] reported that INH-1 knockout mice were protected from isoproterenol-induced cathecolamine injury, whereas INH-1c overexpression [34] resulted in cardiomyomyopathy and susceptibility to lethal arrhythmia. Grote-Wessels et al. [35] reported that heart-specific transgenic overexpression of a constitutively active form of INH-2 failed to rescue pressure overload-induced mice cardiomyopathy. In this regard, our PP1βshRNA approach may attract similar criticism. However, as discussed in the recent reviews by Wittkopper at al [36], the extent of the PP1 inhibition by a PP1 endogenous inhibitor or PP1βshRNA might be essential to determine whether PP1 inhibition can improve cardiac dysfunction without adverse complications. Our heart failure inducible system worked only during the exacerbation of heart failure, suggesting that appropriate regulation in PP1 activity was critical for the therapeutic activity of cardiac SR-mediated Ca^2+^ upregulation. Although we could not determine whether PP1βshRNA can extend the survival time in MLPKO cardiomyopathic mice, ameliorated cardiac BNP production and interstitial fibrosis may be associated with better outcome. Further study investigating the survival of the animals is clearly needed. In this regard, it should be tested in the other animal model which manifests clinically relevant features of human heart failure.

From the clinical viewpoint, a cardiac gene therapy clinical trial using intracoronary catheter delivery of AAV1 expressing SERCA2a is already underway to test the beneficial effect of SERCA2a supplementation in patients with severe heart failure [37]. The phase 2 study results were very promising [11], but a potential drawback is that it may not be possible to control the gene expression when it no longer be needed. A BNP promoter-driven approach may provide a solution to the critical need for an on/off switch in gene expression, as the healthy heart does not produce BNP.

On the other hand, it is important to be aware of the fact that chronic inotropic therapy is contraindicated for treating patients with chronic heart failure, as earlier clinical trial using milrinone failed to rescue patients, and in fact turned out to cause an adverse outcome in the 1980's [38]. One plausible mechanism for the deleterious effect of this phosphodiesterase III inhibitor was that it was associated with chronic augmentation of intracellular cyclic AMP, and subsequent overactivation of protein kinase A, in the failing hearts [39]. This is unlikely in the PP1 inhibition study, because PP1 inhibition in cardiomyocytes does not increase intracellular cyclic AMP or activate protein kinase A [15]. In this regard, we often face a situation that we have to use an inotropic agent to improve hemodynamic status in patients with acutely exacerbated chronic heart failure. This BNP guided regulatory gene therapy approach targeting PP1β may offer a new strategy that maintains the hemodynamic status only when cardiac function has become acutely exacerbated and there is increased BNP promoter activity in the failing heart.

Regarding the role of AAV serotype 9, it has been reported to be very effective for cardiac gene transfer [22] and stable transgene expression was shown to last more than 1 year in rats. As a higher dose AAV injection has been reported to cause T-cell activation [17], a less immunogenic vector delivery system is required. In this regard, the receptor that recognizes AAV9 was recently determined to be cell surface β-galactose [28], and its transfection efficiency is potentially further modifiable by administrating drugs. Therefore, AAV9 might be a good choice as a vector in combination with heart failure-specific gene regulation for the treatment of severe heart failure in order to minimize any adverse immune response. Further study will be required to establish the optimal clinically relevant gene transfer vector system.

In summary, we performed AAV9-mediated heart-failure condition specific *in vivo* knockdown of PP1β, which is the PP1 isoform harboring the greatest impact on cardiomyocye SR Ca^2+^ uptake via PLN phospholylation, resulting in improved cardiac function and prevention of adverse LV remodeling in the failing heart. Future study using other heart failure animal models, including large animals, is warranted.

## Supporting Information

Figure S1 Immunoblottings of cardiac troponin I, SERCA2a, and PP1 catalytic subunit α and γ in LV homegenates from AdV-transfected mice heart. LV specimens were obtained at 7 days after direct adenoviral injection into the heart.
(TIF)

Figure S2 **A**: Body weight, heart weight, and heart/lung weight ratio in 4 month-old MLP knockout mice and age-matched wild-type littermates. **B**: Immunoblottings of PP1 catalytic subunit isoforms, α, β/δ, and γ in LV homogenates. α-Actininn was used as protein loading control. Left graphs indicates quantitative immunoblot analysis of PP1 α, β/δ, and γ in MLP knockout mice and age-matched wild type littermates.
(TIF)

Figure S3 BNP expression analysis by using real-time RT-PCR in 4–5 month-old MLP knockout mice and age-matched wild type littermate hearts. “*” indicates p<0.05 vs. wild type littermates.
(TIF)

Figure S4 Immunoblottings of cardiac troponin I, SERCA2a, and PP1 catalytic subunit α and γ in LV homegenates from AAV9-transfected mice heart. LV specimens were obtained at 3 month after tail-vein-mediated AAV9 gene transfer.
(TIF)

Table S1 **Sequence information for shRNA and real-time PCR analysis.** The bold characters in the mouse PP1βshRNA and mouse NCshRNA indicate 5′-overhang sequence for the directed ligation reaction with pcDNA6.2-GW/EmGFP-miR plasmid.
(DOC)

Table S2 **Serial echocardiogram data after AdV vector injection.** Abbreviations in the table are as follows; GT: gene transfer, %FS: % fractional shortening of the left ventricle, LVDd: left ventricular end-diastolic dimension, LVDs: left ventricular end-systolic dimension, LVPWs: left ventricular posterior wall thickness at systole. “*” indicates p<0.01 vs. NC-shRNA group (Post GT), n = 14 in each group.
(DOC)
