# Supplementary figures and images for: Heart Failure-Inducible Gene Therapy Targeting Protein Phosphatase 1 Prevents Progressive Left Ventricular Remodeling
Source: PLoS One. 2012 Apr 27;7(4):e35875. doi: 10.1371/journal.pone.0035875 (PMC3338799; doi:10.1371/journal.pone.0035875)

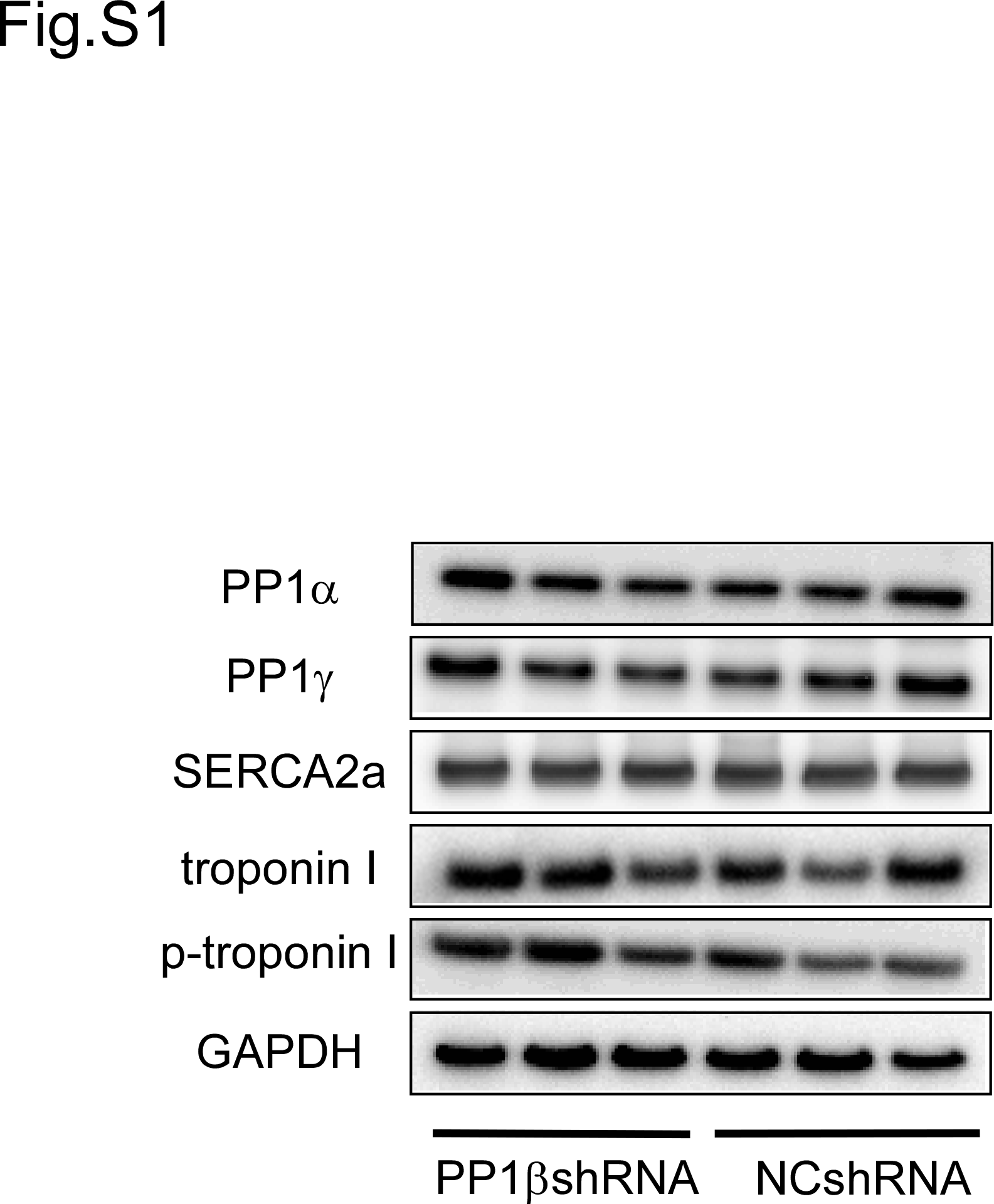

Supplement: Figure S1 — Immunoblottings of cardiac troponin I, SERCA2a, and PP1 catalytic subunit α and γ in LV homegenates from AdV-transfected mice heart. LV specimens were obtained at 7 days after direct adenoviral injection into the heart. (TIF) [file pone.0035875.s001.tif]

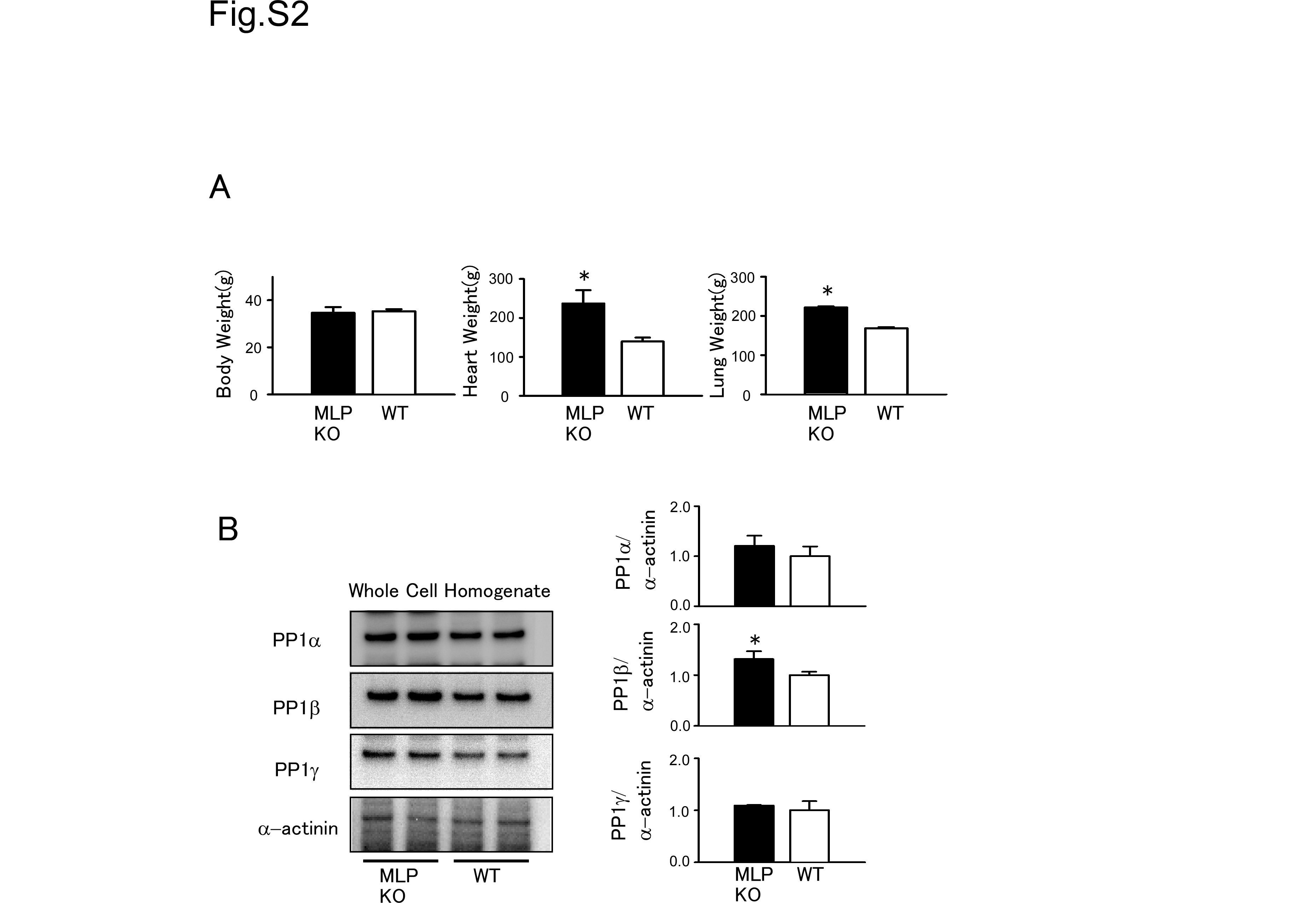

Supplement: Figure S2 — A: Body weight, heart weight, and heart/lung weight ratio in 4 month-old MLP knockout mice and age-matched wild-type littermates. B: Immunoblottings of PP1 catalytic subunit isoforms, α, β/δ, and γ in LV homogenates. α-Actininn was used as protein loading control. Left graphs indicates quantitative immunoblot analysis of PP1 α, β/δ, and γ in MLP knockout mice and age-matched wild type littermates. (TIF) [file pone.0035875.s002.tif]

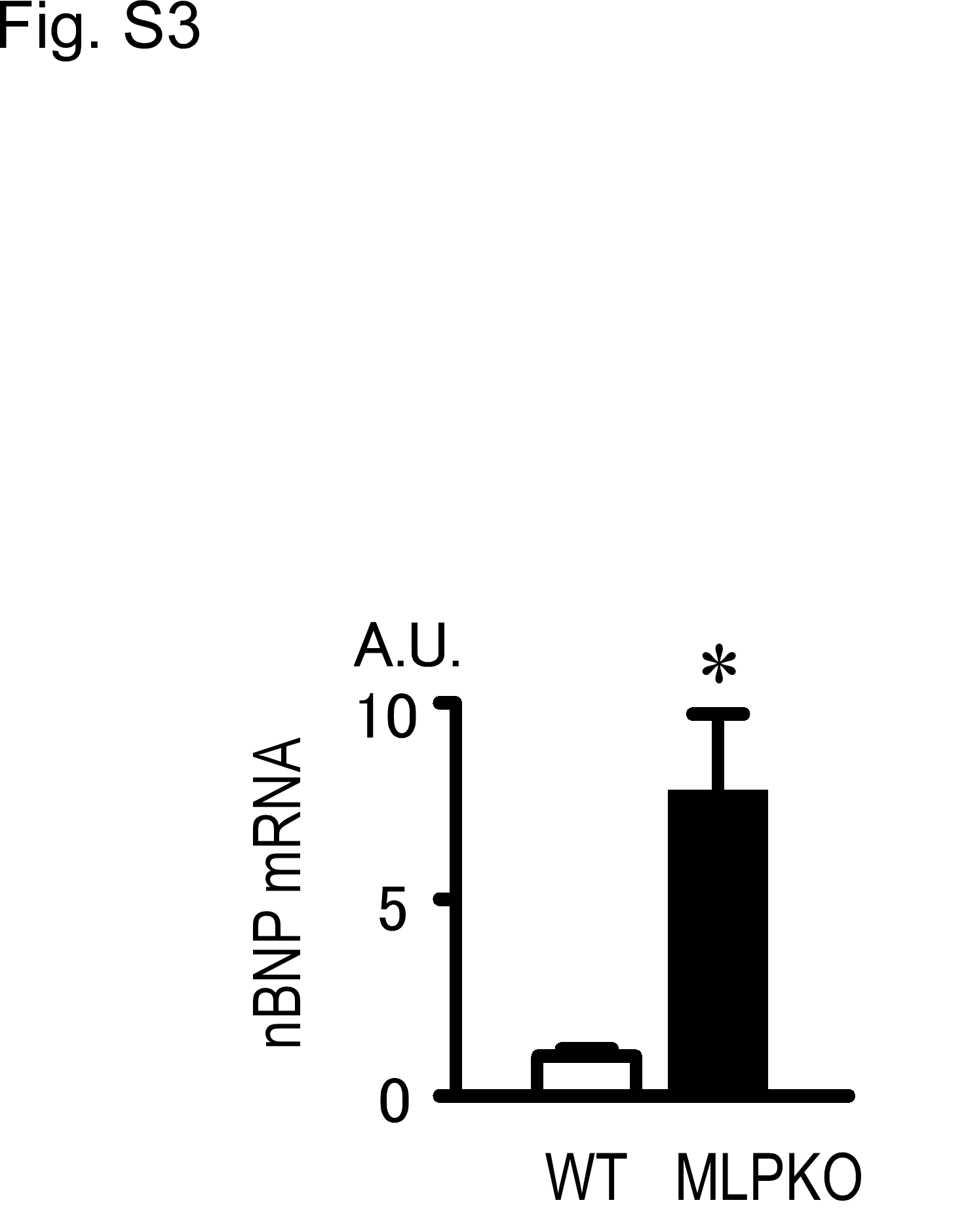

Supplement: Figure S3 — BNP expression analysis by using real-time RT-PCR in 4–5 month-old MLP knockout mice and age-matched wild type littermate hearts. “*” indicates p<0.05 vs. wild type littermates. (TIF) [file pone.0035875.s003.tif]

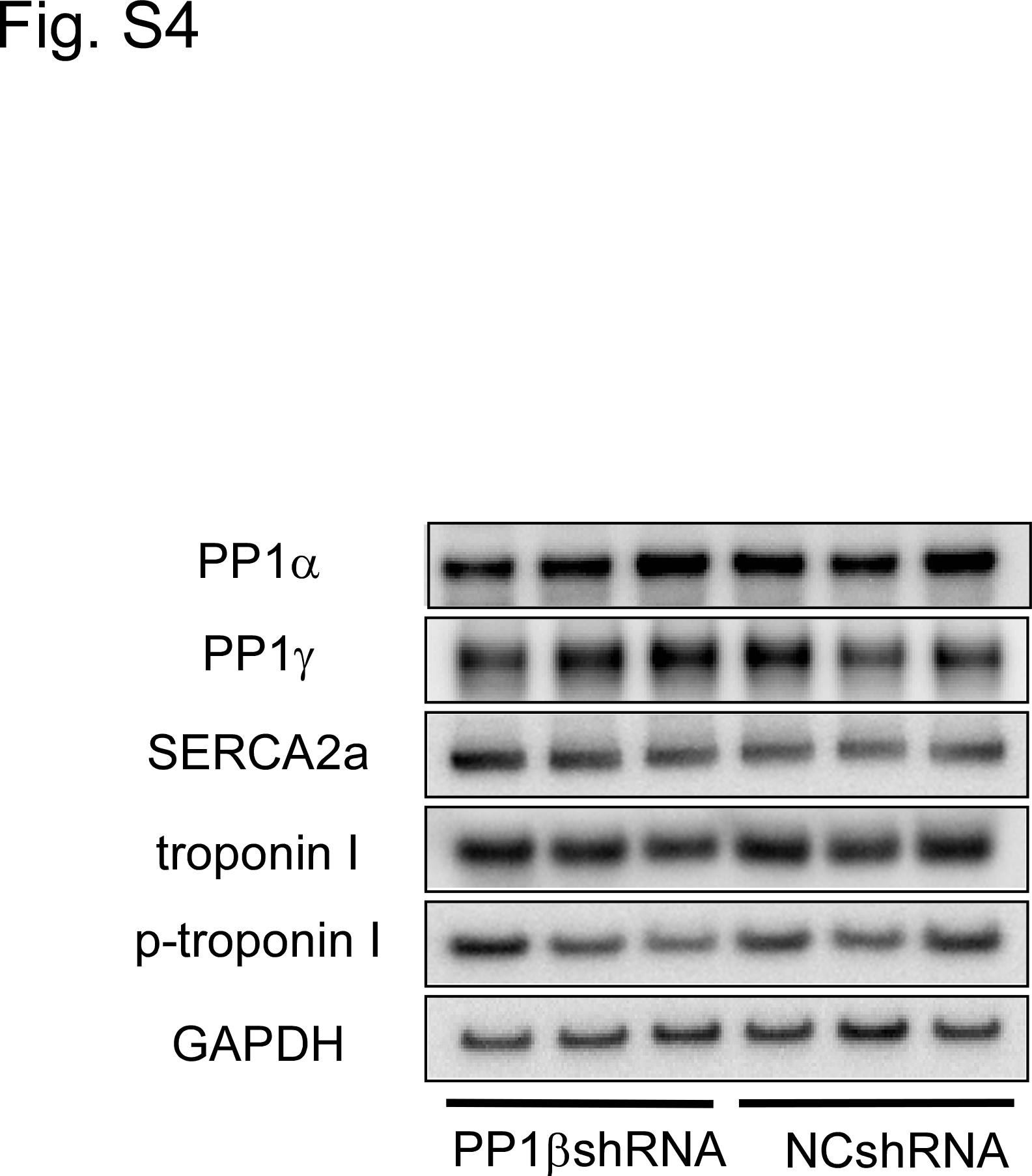

Supplement: Figure S4 — Immunoblottings of cardiac troponin I, SERCA2a, and PP1 catalytic subunit α and γ in LV homegenates from AAV9-transfected mice heart. LV specimens were obtained at 3 month after tail-vein-mediated AAV9 gene transfer. (TIF) [file pone.0035875.s004.tif]
