# Supplementary material for: Heart Failure-Inducible Gene Therapy Targeting Protein Phosphatase 1 Prevents Progressive Left Ventricular Remodeling
Source: PLoS One. 2012 Apr 27;7(4):e35875. doi: 10.1371/journal.pone.0035875 (PMC3338799; doi:10.1371/journal.pone.0035875)
Supplement: Table S1 — Sequence information for shRNA and real-time PCR analysis. The bold characters in the mouse PP1βshRNA and mouse NCshRNA indicate 5′-overhang sequence for the directed ligation reaction with pcDNA6.2-GW/EmGFP-miR plasmid. (DOC) [file pone.0035875.s005.doc]

**Table S1**

|  | Forward | Reverse |
| --- | --- | --- |
| Mouse PP1βshRNA | 5’-**TGCT**GACCACAGTAAGTCACAAAG  CAGTTTTGGCCACTGACTGACTGCTTT  GTCTTACTGTGGT-3’ | 5’-**CCTG**ACCACAGTAAGACAAAGCAGTCA  GTCAGTGGCCAAAACTGCTTTGTGACTTAC  TGTGGTC-3’ |
| Mouse NCshRNA | 5’-**TGCT**GAAATGTACTGCGCGTGGAGACGT  TTTGGCCACTGACTGACGTCTCCACGCAGT  ACATTT-3’ | 5’-**CCTG**AAATGTACTGCGTGGAGACGTCAG  TCAGTGGCCAAAACGTCTCCACGCGCAGTA  CATTTC-3’ |
| Mouse PP1β | 5’-TGAACGTGG ACAGCCTCATC-3’ | 5’-CACGAGACTTGATACACAACCC-3’ |
| Mouse GAPDH | 5'-TGCGGAAGAAAACTGCCTGG-3' | 5'-CGGCTTGGTAAGAAGTCAGACG-3' |
| Mouse BNP | 5’-AAAGTCGGAGGAAATGGCCCAG-3’ | 5’-CG GTCTATCTTGTGCCCAAAGC-3’ |
