# Supplementary material for: Heart Failure-Inducible Gene Therapy Targeting Protein Phosphatase 1 Prevents Progressive Left Ventricular Remodeling
Source: PLoS One. 2012 Apr 27;7(4):e35875. doi: 10.1371/journal.pone.0035875 (PMC3338799; doi:10.1371/journal.pone.0035875)
Supplement: Table S2 — Serial echocardiogram data after AdV vector injection. Abbreviations in the table are as follows; GT: gene transfer, %FS: % fractional shortening of the left ventricle, LVDd: left ventricular end-diastolic dimension, LVDs: left ventricular end-systolic dimension, LVPWs: left ventricular posterior wall thickness at systole. “*” indicates p<0.01 vs. NC-shRNA group (Post GT), n = 14 in each group. (DOC) [file pone.0035875.s006.doc]

**Table S2**

|  | **pre GT** | | **Post GT (1 week)** | |
| --- | --- | --- | --- | --- |
|  | **NCshRNA** | **PP1βshRNA** | **NCshRNA** | **PP1βshRNA** |
| **Body Weight(g)** | **24.8 ± 0.72** | **25.0 ± 0.90** | **24.5±0.61** | **23.5±1.08** |
| **Heart Rate** | **367 ± 5.53** | **369 ± 5.74** | **366±5.74** | **368±6.82** |
| **%FS** | **45.8 ± 1.95** | **45.2 ± 1.19** | **41.9±1.33** | **48.9±1.20** * |
| **LVDd(mm)** | **4.0 ± 0.08** | **4.0 ± 0.08** | **3.9±0.04** | **3.9±0.06** |
| **LVDd(mm)** | **2.2 ± 0.09** | **2.2 ± 0.07** | **2.2±0.06** | **2.0±0.05** |
| **LVPWs(mm)** | **1.1 ± 0.08** | **1.1 ± 0.08** | **1.0±0.04** | **1.2±0.06** |
